# Dopamine in major depressive disorder: A systematic review and meta-analysis of in vivo imaging studies

**DOI:** 10.1177/02698811231200881

**Published:** 2023-10-09

**Authors:** Yuya Mizuno, Abhishekh Hulegar Ashok, Bhagyashree Bhaskar Bhat, Sameer Jauhar, Oliver D Howes

**Affiliations:** 1 Department of Psychosis Studies, Institute of Psychiatry, Psychology & Neuroscience, King’s College London, London, UK; 2 South London and Maudsley NHS Foundation Trust, London, UK; 3 Department of Neuropsychiatry, Keio University School of Medicine, Tokyo, Japan; 4 Psychiatric Imaging Group, MRC London Institute of Medical Sciences, Imperial College London, London, UK; 5 Department of Radiology, University of Cambridge, Cambridge, UK; 6 Department of Radiology, Addenbrooke’s Hospital, Cambridge University Hospitals NHS Foundation Trust, Cambridge, UK; 7 Cambridge and Peterborough NHS Foundation Trust, Cambridge, UK; 8 Department of Psychological Medicine, Institute of Psychiatry, Psychology & Neuroscience, King’s College London, London, UK; 9 Psychiatric Imaging Group, Institute of Clinical Sciences, Faculty of Medicine, Imperial College London, London, UK

**Keywords:** Aetiology, mood, affective disorder, brain, neurochemical, symptoms

## Abstract

**Background:**

Major depressive disorder (MDD) is a leading cause of global disability. Several lines of evidence implicate the dopamine system in its pathophysiology. However, the magnitude and consistency of the findings are unknown. We address this by systematically reviewing in vivo imaging evidence for dopamine measures in MDD and meta-analysing these where there are sufficient studies.

**Methods:**

Studies investigating the dopaminergic system using positron emission tomography or single photon emission computed tomography in MDD and a control group were included. Demographic, clinical and imaging measures were extracted from each study, and meta-analyses and sensitivity analyses were conducted.

**Results:**

We identified 43 studies including 662 patients and 801 controls. Meta-analysis of 38 studies showed no difference in mean or mean variability of striatal D_2/3_ receptor availability (*g* = 0.06, *p* = 0.620), or combined dopamine synthesis and release capacity (*g* = 0.19, *p* = 0.309). Dopamine transporter (DAT) availability was lower in the MDD group in studies using DAT selective tracers (*g* = −0.56, *p* = 0.006), but not when tracers with an affinity for serotonin transporters were included (*g* = −0.21, *p* = 0.420). Subgroup analysis showed greater dopamine release (*g* = 0.49, *p* = 0.030), but no difference in dopamine synthesis capacity (*g* = −0.21, *p* = 0.434) in the MDD group. Striatal D_1_ receptor availability was lower in patients with MDD in two studies.

**Conclusions:**

The meta-analysis indicates striatal DAT availability is lower, but D_2/3_ receptor availability is not altered in people with MDD compared to healthy controls. There may be greater dopamine release and lower striatal D_1_ receptors in MDD, although further studies are warranted. We discuss factors associated with these findings, discrepancies with preclinical literature and implications for future research.

## Introduction

Major depressive disorder (MDD) is common, affects 10% of the population and is a leading cause of global disability (Hasin et al., 2018). Dopamine dysregulation is hypothesised to underlie some core depressive symptoms, such as anhedonia and psychomotor disturbance (Belujon and Grace, 2017; Di Chiara et al., 1999; Dunlop and Nemeroff, 2007; Nestler and Carlezon, 2006; Nutt, 2006). Three lines of preclinical investigation suggest a role for dopaminergic alterations in depression. First, the chronic mild stress animal model of depression has shown reduced dopamine neuron activity (Chang and Grace, 2014). Second, animals with lesions in the ventral tegmental area (VTA), one of the main origins of dopamine neurons in the brain, show learned helplessness behaviour (a feature of depression) (Winter et al., 2007). Third, selective optical stimulation of dopamine neurons in the VTA induces resilience in animals that exhibit depression-like behaviour, as measured by social avoidance and sucrose preference tests (Chaudhury et al., 2013; Muir et al., 2019).

In the clinical literature, meta-analyses have reported improvements in depressive symptoms after treatment with dopamine receptor partial agonists such as aripiprazole (Strawbridge et al., 2019) and cariprazine (Romeo et al., 2018), as well as the dopamine agonist pramipexole (Tundo et al., 2019). Consequently, some authors have speculated that patients with MDD show an underlying dopaminergic abnormality (Hori and Kunugi, 2013). This preclinical and clinical evidence provides indirect evidence for a possible role of dopamine in MDD. Direct evidence can be derived from positron emission tomography (PET) and single photon emission computed tomography (SPECT) studies, which measure dopaminergic function in vivo in humans (Kim et al., 2013). Using these techniques, several studies have investigated dopamine synthesis (Agren et al., 1993; Martinot et al., 2001; Wing et al., 2015), dopamine release (Busto et al., 2009; Parsey et al., 2001; Schneier et al., 2018), dopamine transporter (DAT; Amsterdam et al., 2012; Argyelan et al., 2005; Brunswick et al., 2003; Camardese et al., 2014; Hellwig et al., 2018; Hsiao et al., 2013; Hsieh et al., 2010; Laasonen-Balk et al., 1999; Lehto et al., 2006, 2008b; Malison et al., 1998; Meyer et al., 2001; Moriya et al., 2020; Pizzagalli et al., 2019; Sarchiapone et al., 2006; Wu et al., 2011; Yang et al., 2008) and dopamine D_2/3_ receptor (Brody et al., 2009; Busto et al., 2009; De Kwaasteniet et al., 2014; D’Haenen and Bossuyt, 1994; Ebert et al., 1996; Hamilton et al., 2018; Hirvonen et al., 2008; Klimke et al., 1999; Kuroda et al., 2006; Messa et al., 2003; Meyer et al., 2006; Montgomery et al., 2007; Moses-Kolko et al., 2012; Parsey et al., 2001; Pecina et al., 2017; Savitz et al., 2013; Schneier et al., 2018; Shah et al., 1997; Wing et al., 2015; Yang et al., 2008) availability in MDD. However, the magnitude and consistency of findings across studies are unclear, and to our knowledge, there has not been a previous systematic review and meta-analysis of dopamine synthesis, release and D_2/3_ receptor findings. Thus, we aimed to synthesise the PET and SPECT imaging findings on dopaminergic function in MDD and to consider their implications for its treatment. We grouped studies into those examining striatal dopamine receptor availability, DAT availability and presynaptic dopamine function (i.e. dopamine synthesis capacity and dopamine release). We focused on the striatum as it is richly innervated with dopaminergic neurons and reliably imaged with PET and SPECT in humans (Kanthan et al., 2017).

## Methods

This study was registered with the PROSPERO international prospective register of systematic reviews (CRD42022300529) (Booth et al., 2012), and adhered to the PRISMA 2020 statement for reporting systemic reviews (Supplemental Table 1) (Page et al., 2021).

### Search strategy

EMBASE, Medline, PsycINFO, ClinicalTrials.gov and Cochrane CENTRAL databases were searched from inception date to 24th August 2022 for relevant articles without language restrictions. The electronic searches using EMBASE, Medline and PsycINFO were carried out together using Ovid. The following keywords were used: (Positron Emission Tomography OR PET OR Single photon emission tomography OR SPET OR Single Photon Emission Computed Tomography OR SPECT) AND (dopamine OR dopamine release OR dopamine synthesis OR dopamine availability OR dopamine transporter OR dopamine reuptake OR dopamine receptor) AND (MDD OR major depressive disorder OR depression). The reference lists of included studies and relevant review articles were manually searched for additional studies.

### Inclusion and exclusion criteria

To be included in the meta-analysis, articles needed to report dopaminergic function in MDD and a control group, including the mean and standard deviation values for both groups. We included (1) original molecular imaging studies that indexed striatal dopamine D_2/3_ receptors, DATs, dopamine synthesis, dopamine release and dopamine D_1_ receptors, (2) studies conducted in people with a diagnosis of MDD and (3) studies reporting data for the whole striatum or a striatal subregion. We also included studies examining extra-striatal dopamine availability and summarised these separately. We excluded studies that did not have a healthy control group, primarily focused on people with a diagnosis of bipolar affective disorder or other mood disorders, included participants with significant neuropsychiatric or physical comorbidities, or recruited participants under 18 years of age. For studies with overlapping participants, we chose the most relevant study, or included the study with the largest sample size and excluded the smaller study from the meta-analysis to avoid duplication of participants.

### Study selection and data extraction

Two researchers (YM and AA) independently searched and identified studies meeting the inclusion criteria. The two researchers then used standardised spreadsheets to independently extract the following data: name of the first author, year of publication, number of included patients and controls, age and sex of participants, diagnostic criteria used for MDD, clinical variables (i.e. the presence of psychotropic treatment, mention of treatment-resistance, depression rating scales), imaging characteristics (i.e. PET or SPECT, type of radiotracer used, regions of interest, reference region, method of drug challenge for studies of dopamine release) and results of dopaminergic indices as mean and standard deviation values. Any disagreement during study selection and data extraction was resolved through discussion with a third researcher (OH). Where data regarding dopaminergic indices were unreported, we contacted the corresponding authors to obtain additional information.

### Risk of bias assessment

Two researchers (YM and BBB) independently assessed the risk of bias (quality) of included studies using the Newcastle–Ottawa Scale for case–control studies (Wells et al., 2000). We rated study-level risk of bias based on three categories (i.e. Selection, Comparability, Exposure) and used the star system to summarise these findings. For Comparability, we assessed whether studies explicitly matched patient and control groups for age and sex in their study design, or adjusted for these in their analysis, as these variables are known to influence dopaminergic indices (Lavalaye et al., 2000; Malen et al., 2022). We examined the association between overall quality ratings of individual studies in the Newcastle–Ottawa Scale and effect sizes in the meta-analysis.

### Outcome measures

Our primary outcomes were standardised mean differences (Hedges’ *g*) in dopamine imaging measures in the whole striatum between people with MDD and controls. As a secondary outcome, we calculated the coefficient of variation ratio (CVR) to examine differences in the variability of D_2/3_ receptor and DAT availability across groups (Senior et al., 2020). A CVR value of 1 indicates equal relative variability in dopaminergic measures in the MDD and control groups, with greater and smaller CVR values representing greater and smaller variability in the MDD group, respectively (Brugger and Howes, 2017). For the meta-analysis of variability, we selected CVR as our outcome measure to account for the mean scaling of variability (Eisler et al., 2008). Other secondary outcomes included the effects of age and depression severity (as measured by the Hamilton Depression Rating Scale 17-item version (HAM-D) (Hamilton, 1960)) on D_2/3_ receptor and DAT availability. Where depression severity was reported using the Beck Depression Inventory (Beck et al., 1961), Montgomery-Åsberg Depression Rating Scale (Montgomery and Asberg, 1979) or the Quick Inventory of Depressive Symptomatology (Rush et al., 2003), we used previously reported conversion tables to calculate HAM-D equivalent scores (Furukawa et al., 2019; Leucht et al., 2018; Rush et al., 2003).

### Meta-analysis

Where only values of striatal subdivisions were reported, we averaged the striatal subdivision values to estimate the value for the whole striatum, as described in other imaging meta-analyses (Howes et al., 2012; Kambeitz et al., 2014). The Statstodo software was used for the estimation of pooled standard deviation (http://statstodo.com/ComMeans_Pgm.php). The Plot Digitizer software was used to extract data from studies where data were only available in a plot format (http://plotdigitizer.sourceforge.net/). The statistical analysis of the extracted data was conducted using the R statistical programming language version 3.6.1 with the ‘metafor’ package. We required a minimum of five studies to carry out quantitative syntheses, as findings from meta-analyses with a small number of studies may be less reliable (Fu et al., 2011). Meta-analysis was carried out using a random effects model, and findings were visually summarised using forest plots. Meta-regression was carried out to assess the moderating effects of age and depression severity on effect sizes in studies of D_2/3_ receptor and DAT availability. Pre-planned subgroup analyses were carried out for D_2/3_ receptor and DAT studies to examine the effects of radiotracers used, drug-naïve status and treatment-resistance in MDD cohorts. As an exploratory analysis, we grouped studies examining dopamine synthesis capacity and dopamine release to meta-analyse presynaptic dopamine function (Howes et al., 2012). Studies examining dopamine D_1_ receptors and extra-striatal dopamine availability were summarised qualitatively.

Heterogeneity was estimated using the *I*^2^ value (*I*^2^ < 50% indicates low to moderate heterogeneity, whereas *I*^2^ > 50% indicates moderate to high heterogeneity). Leave-one-out analyses were carried out to examine the consistency of findings in meta-analyses of mean difference. Publication bias was assessed by visually inspecting funnel plots and carrying out regression tests to explore the number of missing negative studies. A significance level of *p* < 0.05 (two-tailed) was taken as significant for all analyses.

### Post hoc analysis

Treatment with serotonin reuptake inhibitors, by increasing extracellular serotonin levels, may have agonist effects on midbrain 5-HT_2A_ receptors, thereby increasing the firing of dopamine neurons in the striatum (Dremencov et al., 2009). Radioligands for D_2/3_ receptors are known to be sensitive to alterations in dopamine release due to competition with endogenous dopamine when binding to post-synaptic receptors (Laruelle, 2000). Accordingly, the use of serotonin reuptake inhibitors may confound results for striatal D_2/3_ receptor availability. To address this, we carried out a post hoc analysis of D_2/3_ receptor availability in patients who were currently not on serotonin reuptake inhibitors. Finally, we excluded studies that used [^123^I]B-CIT or [^99m^Tc]TRODAT-1 and meta-analysed DAT availability in the remaining studies, as these radiotracers are known to have poor selectivity between dopamine and serotonin transporters (Dresel et al., 1999; Laruelle et al., 1993).

## Results

In all, 43 studies met the inclusion criteria for the systematic review, of which 38 studies were included in the meta-analysis (Supplemental Figure 1). The characteristics of the included studies and their main findings are summarised in Supplemental Tables 2–7. The risk of bias assessment using the Newcastle–Ottawa Scale showed that most studies had a ‘Good’ or ‘Fair’ overall quality rating, while four studies were rated as ‘Poor’ (Agren et al., 1993; D’Haenen & Bossuyt, 1994; Dubol et al., 2020; Wu et al., 2011) (Supplemental Table 8). Importantly, 88% of included studies explicitly matched MDD and control groups for age in their study design, or adjusted for this confounder in their analysis (see Supplemental Table 8 for details).

### Dopamine receptor availability

**Figure 1.**
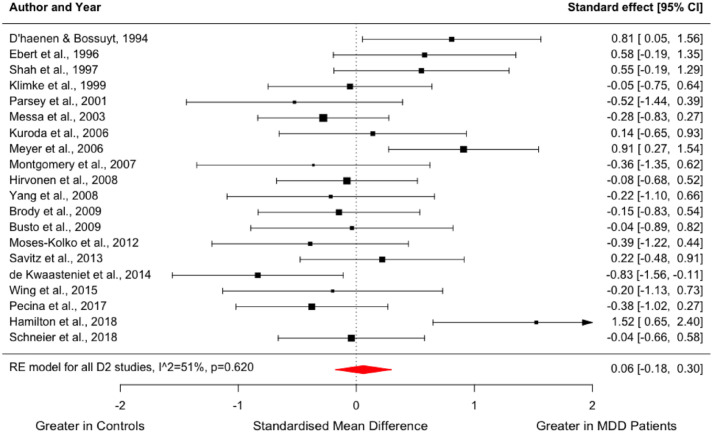
Forest plot showing effect sizes for D_2/3_ receptor availability in MDD. The forest plot shows effect sizes using a random-effects model, with 95% CIs for striatal D_2/3_ receptor availability. There was no significant difference in striatal dopamine receptor availability in people with MDD compared to controls (Hedges’ *g* = 0.06; 95% CI, −0.18–0.30, *p* = 0.620). CI: confidence interval; MDD: major depressive disorder.

In all, 20 studies compared dopamine D_2/3_ receptor availability between 309 people with MDD and 334 controls (Brody et al., 2009; Busto et al., 2009; De Kwaasteniet et al., 2014; D’Haenen and Bossuyt, 1994; Ebert et al., 1996; Hamilton et al., 2018; Hirvonen et al., 2008; Klimke et al., 1999; Kuroda et al., 2006; Messa et al., 2003; Meyer et al., 2006; Montgomery et al., 2007; Moses-Kolko et al., 2012; Parsey et al., 2001; Pecina et al., 2017; Savitz et al., 2013; Schneier et al., 2018; Shah et al., 1997; Wing et al., 2015; Yang et al., 2008). The meta-analysis revealed no significant difference in striatal D_2/3_ receptor availability in MDD relative to controls with a summary effect size of 0.06 (95% confidence interval (CI), −0.18–0.30, *p* = 0.620, Figure 1).

*I*^2^ was 51% for the meta-analysis of dopamine D_2/3_ receptor availability, indicating moderate heterogeneity. The summary effect size did not change significantly in the leave-one-out analysis, with Hedges’ *g* varying from 0.00 to 0.11 (all *p* > 0.05). Visual inspection of the funnel plot and regression test did not indicate publication bias (Supplemental Figure 2).

**Figure 2.**
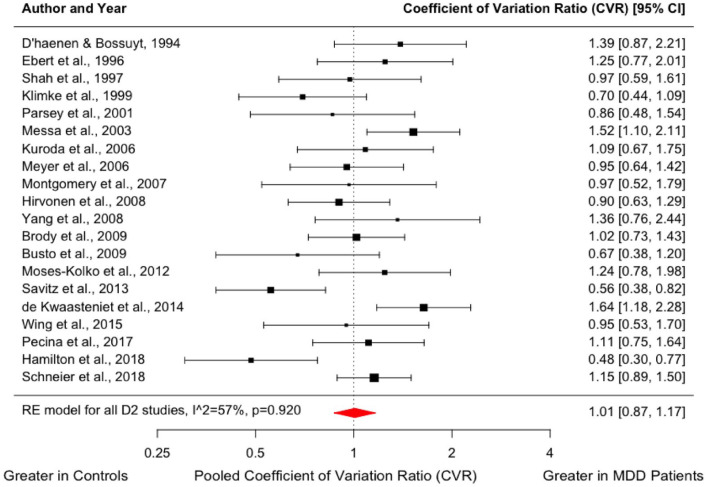
Forest plot showing effect sizes for CVR of D_2/3_ receptor availability in MDD. There was no difference in the variability of striatal dopamine receptor availability in the MDD group compared to healthy volunteers (CVR = 1.01; 95% CI, 0.87–1.17, *p* = 0.920). CVR: coefficient of variation ratio; CI: confidence interval; MDD: major depressive disorder.

We found no difference in the variability of dopamine receptor availability in the MDD group compared to healthy controls (CVR = 1.01; 95% CI, 0.87–1.17, *p* = 0.920, Figure 2). Meta-regression did not indicate a significant effect of the mean age of patients (*z* = −1.211; *p* = 0.226) or mean HAM-D equivalent scores (*z* = 0.310; *p* = 0.756) on dopamine D_2/3_ availability (Supplemental Figure 3).

### DAT availability

**Figure 3.**
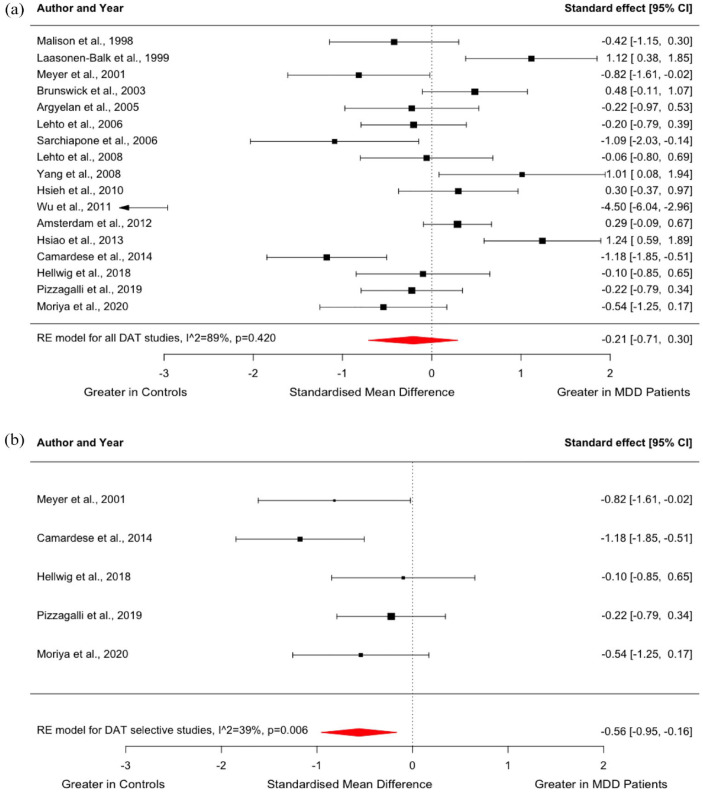
Forest plot showing effect sizes for DAT availability in MDD: (a) Effect sizes for all studies assessing DAT availability. The forest plot shows effect sizes using a random-effects model, with 95% CIs for striatal DAT availability. There was no significant difference in DAT availability in people with MDD compared to controls (Hedges’ *g* = −0.21; 95% CI, −0.71 to 0.30, *p* = 0.420), and (b) effect sizes for studies using radiotracers with high selectivity for DATs. The forest plot shows effect sizes using a random-effects model, with 95% CIs for striatal DAT availability. When studies using [^123^I]B-CIT or [^99m^Tc]TRODAT-1 were excluded, DAT availability was significantly lower in people with MDD compared to controls (Hedges’ *g* = −0.56; 95% CI, −0.95 to −0.16, *p* = 0.006). CIs: confidence intervals; DAT: dopamine transporter; MDD: major depressive disorder.

A total of 17 studies were identified, comparing DAT availability between people with MDD (*n* = 291) and controls (*n* = 392) (Amsterdam et al., 2012; Argyelan et al., 2005; Brunswick et al., 2003; Camardese et al., 2014; Hellwig et al., 2018; Hsiao et al., 2013; Hsieh et al., 2010; Laasonen-Balk et al., 1999; Lehto et al., 2006; Lehto et al., 2008b; Malison et al., 1998; Meyer et al., 2001; Moriya et al., 2020; Pizzagalli et al., 2019; Sarchiapone et al., 2006; Wu et al., 2011; Yang et al., 2008). Meta-analysis indicated no significant difference in striatal DAT availability in MDD relative to controls with an effect size of −0.21 (95% CI, −0.71–0.30, *p* = 0.420, Figure 3(a)).

*I*^2^ of 89% indicated high heterogeneity across studies in the meta-analysis of DAT availability. This high heterogeneity was mainly due to the study by Wu et al. (2011), which had a ‘Poor’ overall quality rating on the Newcastle-Ottawa Scale, and demonstrated lower DAT availability in 13 patients with MDD relative to 10 controls with an effect size of 4.50 (Wu et al., 2011). Nevertheless, the summary effect size did not differ significantly in the leave-one-out analysis, with Hedges’ *g* varying from −0.29 to −0.02 (all *p* > 0.05). Visual inspection of the funnel plot and regression test suggested publication bias (*z* = −3.48, *p* ⩽ 0.001), with small studies with a large positive effect size possibly being unpublished (Supplemental Figure 4). However, the asymmetry in the funnel plot was largely due to the study by Wu et al. (2011) which reported a large negative effect size.

**Figure 4.**
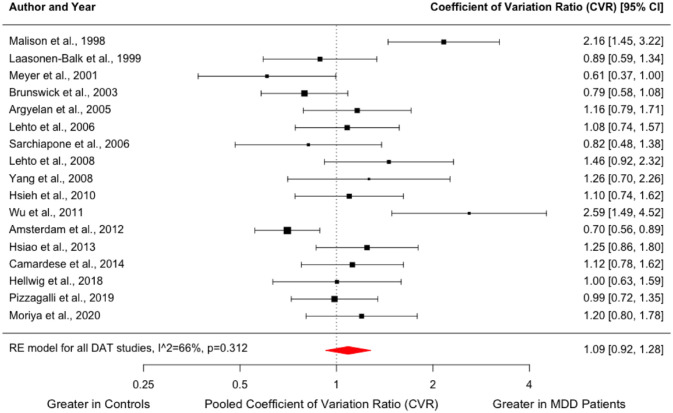
Forest plot showing effect sizes for CVR of DAT availability in MDD. There was no difference in the variability of striatal DAT availability in the MDD group compared to healthy volunteers (CVR = 1.09; 95% CI, 0.92–1.28, *p* = 0.312). CI: confidence interval; CVR: coefficient of variation ratio; DAT: dopamine transporter; MDD: major depressive disorder.

Similar to D_2/3_ receptor studies, we found no significant difference in the variability of DAT availability in the MDD group compared to controls (CVR = 1.09; 95% CI, 0.92–1.28, *p* = 0.312, Figure 4). Meta-regression indicated no influence of the mean age of patients (*z* = −1.103; *p* = 0.270) or mean HAM-D scores (*z* = −0.052; *p* = 0.959) on DAT availability (Supplemental Figure 5).

### Pre-planned subgroup analysis for dopamine receptor and transporter availability

Pre-planned subgroup analyses were carried out where three or more studies were available per subgroup (Supplemental Table 9). The null findings regarding D_2/3_ receptor availability remained the same when studies using [^11^C]raclopride (*g* = 0.11; 95% CI, −0.23 to 0.45, *p* = 0.516) and [^123^I]IBZM (*g* = 0.06; 95% CI, −0.41 to 0.52, *p* = 0.813) were examined separately. Regarding clinical subpopulations, only two studies examined D_2/3_ receptor availability in drug-naïve patients with MDD (Hirvonen et al., 2008; Schneier et al., 2018), and only two studies in patients with treatment-resistant depression (De Kwaasteniet et al., 2014; Kuroda et al., 2006). All four studies did not find a significant difference in D_2/3_ receptor availability between patients and controls, and excluding these studies from the main analysis did not yield different findings. Similarly, pre-planned subgroup analyses for studies of DAT availability did not show significant differences in subgroups (Supplemental Table 9).

### Post hoc analysis for dopamine receptor and transporter availability

To account for the sensitivity of dopamine receptor imaging methods to alterations in endogenous dopamine levels, we meta-analysed striatal D_2/3_ receptor availability in patients who were currently not on serotonin reuptake inhibitors. Nine and two studies measured D_2/3_ receptor availability in drug-free patients (Busto et al., 2009; D’Haenen and Bossuyt, 1994; Klimke et al., 1999; Meyer et al., 2006; Moses-Kolko et al., 2012; Parsey et al., 2001; Pecina et al., 2017; Savitz et al., 2013; Yang et al., 2008) and drug-naïve patients (Hirvonen et al., 2008; Schneier et al., 2018), respectively. Moreover, two studies reported data on a subgroup of patients who were drug-free (Brody et al., 2009) and drug-naïve (Messa et al., 2003). Pooling data from these 13 studies, striatal D_2/3_ receptor availability did not differ between 201 drug-free patients with MDD and 238 healthy controls (Hedges’ *g* = −0.15; 95% CI, −0.53 to 0.22, *p* = 0.428) (Supplemental Figure 6). *I*^2^ of this meta-analysis was 71%, indicating high heterogeneity.

In addition, we carried out a post hoc analysis excluding studies that used [^123^I]B-CIT or [^99m^Tc]TRODAT-1 to measure striatal DAT availability. Using radiotracers with high selectivity for DATs, five studies compared DAT availability between people with MDD (*n* = 81) and healthy controls (*n* = 105) (Camardese et al., 2014; Hellwig et al., 2018; Meyer et al., 2001; Moriya et al., 2020; Pizzagalli et al., 2019). Interestingly, this meta-analysis showed significantly lower striatal DAT binding in the patient group (Hedges’ *g* = −0.56; 95% CI, −0.95 to −0.16, *p* = 0.006) (Figure 3(b)). *I*^2^ of this meta-analysis was 39%, indicating low heterogeneity.

### Presynaptic dopamine function

**Figure 5.**
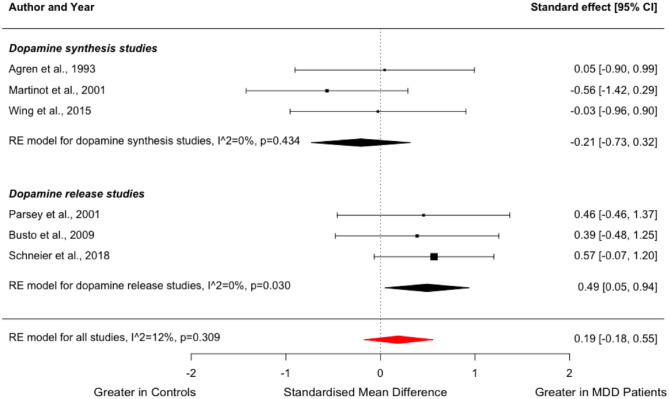
Forest plot showing effect sizes for presynaptic dopamine function in MDD. The forest plot shows effect sizes using a random-effects model, with 95% CIs for striatal dopamine synthesis capacity and dopamine release capacity. There was no difference in the summary effect size in people with MDD compared to controls for striatal dopamine synthesis and release capacity combined (Hedges’ *g* = 0.19; 95% CI, −0.18 to 0.55, *p* = 0.309) or for dopamine synthesis capacity on its own (Hedges’ *g* = −0.21; 95% CI, −0.73 to 0.32, *p* = 0.434), but dopamine release capacity was significantly higher in patients with MDD relative to controls (Hedges’ *g* = 0.49; 95% CI, 0.05–0.94). CI: confidence interval; MDD: major depressive disorder.

Three studies compared dopamine synthesis capacity between people with MDD (*n* = 27) and healthy volunteers (*n* = 31) (Agren et al., 1993; Martinot et al., 2001; Wing et al., 2015), while three other studies compared dopamine release capacity in a total of 39 patients and 41 controls (Busto et al., 2009; Parsey et al., 2001; Schneier et al., 2018). The studies on dopamine release used either an oral or intravenous amphetamine challenge. These six studies were grouped for an exploratory meta-analysis to yield a summary effect size for presynaptic dopamine function (Figure 5). While the subgroup of dopamine release studies indicated significantly greater striatal dopamine release in the MDD group (*g* = 0.49; 95% CI, 0.05–0.94, *p* = 0.030), the summary effect size for overall presynaptic striatal dopamine function was not significantly different between patients and controls (*g* = 0.19; 95% CI, −0.18 to 0.55, *p* = 0.309), and there was no significant group difference in the studies of dopamine synthesis capacity (Hedges’ *g* = −0.21; 95% CI, 0.73 to 0.32, *p* = 0.434). This result did not change when excluding a study with a ‘Poor’ overall quality rating in the risk of bias assessment (Agren et al., 1993) (*g* = 0.20; 95% CI, −0.22 to 0.63, *p* = 0.344). None of the studies reporting on dopamine release capacity included patients who were receiving serotonin reuptake inhibitors.

### Other striatal dopaminergic measures

*D_1_ receptor availability*: Two studies assessed D_1_ receptor availability (Supplemental Table 6). Both studies showed lower D_1_ availability in a striatal region in people with MDD, although one was the whole striatum (Dougherty et al., 2006), while the other study reported this in the left middle caudate (Cannon et al., 2009).

*Extra-striatal dopaminergic measures*: One study assessed D_2_ availability in the amygdala, hippocampus, frontal cortex, anterior cingulate gyrus, thalamus, brain stem and cerebellum using [^11^C]FLB 457, but did not detect significant differences between patients with MDD and healthy controls (Montgomery et al., 2007). Similarly, another study with the same tracer did not report a significant difference between groups in the anterior cingulate cortex (Saijo et al., 2010). In a study that assessed D_2_ receptor availability in the temporal cortex using [^123^I]Epidepride, no alterations were seen in people with MDD (Lehto et al., 2008a). Finally, a study using a DAT tracer, [^11^C]PE2I, reported reduced uptake in the superior part of the midbrain including the substantia nigra and VTA of people with MDD compared to controls (Dubol et al., 2020).

## Discussion

Our main findings are that there is no difference in striatal D_2/3_ receptor and transporter availability in MDD compared to healthy controls when all tracers were included, but DAT availability was significantly lower in the MDD group after excluding studies using tracers with appreciable affinity for serotonin transporters. Subgroup analysis of striatal dopamine release studies indicated significantly greater dopamine release in the MDD group, but no alteration in striatal dopamine synthesis capacity. To our knowledge, this is the first meta-analysis investigating striatal D_2/3_ receptor availability or dopamine synthesis and release capacity in vivo in MDD. A previous meta-analysis of 12 DAT studies in depression did not reveal a significant difference between MDD and healthy controls (Li et al., 2015). Our results are consistent with this finding and extend it to a larger cohort of patients, but also importantly, find evidence that DAT is lower in MDD after excluding studies using tracers with appreciable affinity for serotonin transporters. Our sensitivity analyses of dopamine D_2/3_ receptor availability showed consistent results across differing radiotracers and patient cohorts. Together, these data indicate that alterations of striatal dopaminergic receptors in people with MDD are unlikely, but lower striatal DAT availability is associated with MDD. Our systematic review found no evidence for altered D_2/3_ receptor availability in extra-striatal regions either, but some evidence, from two studies for lower striatal D_1_ receptor availability (Cannon et al., 2009; Dougherty et al., 2006), and in one study, lower DAT in the midbrain (Dubol et al., 2020).

Some issues need to be considered while interpreting these findings. Most of the D_2/3_ tracers do not distinguish between D_2_ and D_3_ receptors, or between high- and low-affinity forms of the D_2_ receptor. Thus, it is possible that differences in one of these components could be masked by alterations in other aspects (Brugger et al., 2020; Howes et al., 2012). However, one study used [^11^C]-PHNO, which is selective for the D_2_ high-affinity form and shows higher affinity for D_3_ over D_2_ receptors (Schneier et al., 2018), but did not show statistically significant differences between MDD and controls in the striatum. Thus, there is currently no evidence to suggest there are differences in these components, although further studies are needed to fully exclude this possibility.

### Limitations

We identified moderate to high between-study inconsistency across analyses. In common with meta-analyses of other psychiatric imaging studies (Ashok et al., 2017, 2019; Howes et al., 2012), there are variations between studies in both the sample and imaging characteristics that may contribute to the between-study inconsistency. Nevertheless, the random-effects model we used allows for variations in effects. It should also be noted that there have been a relatively small number of studies that assessed dopamine release, dopamine synthesis, extra-striatal dopamine or D_1_ receptor availability, and sample sizes are small. Thus, conclusions for these measures should be considered preliminary.

A substantial number of studies of DAT used radiotracers shown to have an affinity for both DAT and serotonin transporter (SERT). This limits the interpretation of findings as DAT abnormality may be masked by alteration in SERT level (Li et al., 2015; Pizzagalli et al., 2019). Relevant to this point, our post hoc analysis, which excluded studies using radiotracers with poor selectivity between DAT and SERT, showed that striatal DAT availability is lower in MDD relative to healthy controls. This may indicate a compensatory mechanism for decreased dopamine concentrations in the synaptic cleft, although the sample size is small and should be interpreted with caution. Future studies assessing DAT availability in MDD should use radiotracers selective for DATs, as alterations in the serotonergic system may be common in this patient group.

Smoking cigarettes has been associated with an acute decrease in [^11^C]raclopride binding within the ventral striatum (Brody et al., 2004). Despite this, of the 20 studies included in our meta-analysis of striatal D_2/3_ receptor availability, only four studies excluded participants who were current smokers (Meyer et al., 2006; Pecina et al., 2017; Savitz et al., 2013; Schneier et al., 2018), while another study reported data on a subgroup of patients and healthy controls who were nonsmokers (Busto et al., 2009). This is an important limitation of the literature, as the inclusion of current smokers is expected to add variability to measures of striatal D_2/3_ receptor availability, thereby obscuring the effects of the disease.

The use of the HAM-D as a measure of depression severity has a number of limitations, given its structure, and reliance on measures such as sleep (three items), and other features of depression (Hieronymus et al., 2020). Consequently, the lack of association we report between dopamine D_2/3_ receptor and transporter availability and HAM-D scores may reflect this. Focusing on symptom domains of depression such as anhedonia, which may be more closely associated with dopamine function, may be a promising approach for future studies. Supporting this, Cannon et al. reported that [^11^C]NNC-112 left-to-right BP_ND_ ratio correlated inversely with the Inventory of Depressive Symptoms-Clinician Version anhedonia subscale (Cannon et al., 2009). Furthermore, Peciña and colleagues found a negative correlation between D_2/3_ receptor availability in the bilateral nucleus accumbens and the severity of motivational anhedonia as assessed using the Apathy Evaluation Scale (Pecina et al., 2017). However, other studies did not demonstrate associations between dopaminergic measures and anhedonia. For example, neither striatal D_2/3_ receptor availability nor dopamine release showed a significant association with anhedonia as indexed by the Snaith–Hamilton Pleasure Scale (SHAPS) (Schneier et al., 2018), or as the sum of interest in recreational, sexual and work-related activities in the Scale for the Assessment of Negative Symptoms (Parsey et al., 2001). Furthermore, there was no significant association between DAT availability in the striatum and SHAPS scores (Camardese et al., 2014; Pizzagalli et al., 2019; Sarchiapone et al., 2006). Similarly, there was no association between DAT binding and anhedonia measured as scores of HAM-D Items 7 and 8 (Hsiao et al., 2013). This highlights the need for further research in this area. Likewise, another limitation in the literature is the paucity of studies evaluating associations between D_2/3_ receptor binding in the dorsal putamen and motor retardation using validated neuropsychological measures (Meyer et al., 2006).

Despite indirect pharmacological evidence of dopaminergic abnormality in a subgroup of patients with treatment-resistant MDD (Hori and Kunugi, 2013), only three studies assessed dopaminergic abnormality exclusively in patients with treatment-resistant MDD (De Kwaasteniet et al., 2014; Hellwig et al., 2018; Kuroda et al., 2006). Future studies are needed to address this issue.

### Implications and future research directions:

Our findings of significantly greater striatal dopamine release and lower DAT availability in MDD in the absence of D_2/3_ receptor alterations indicate specific dopamine alterations in this disorder. Whilst both the dopamine release and DAT findings warrant further testing given the relatively small number of studies to date, there is no indication of blunted striatal dopamine release in MDD. The first major implication of our meta-analysis is thus that findings in preclinical models of deficits in dopamine release (Di Chiara and Tanda, 1997) do not reflect what is seen in human studies to date. This discrepancy could reflect the possibility that blunted dopamine release is specific to a sub-group of patients with MDD which may be associated with resistance to serotonergic treatments. Supporting this possibility, ketamine, which has efficacy in patients with treatment-resistant MDD (Conley et al., 2021), increases striatal dopamine levels (Kokkinou et al., 2018) and synthesis capacity (Kokkinou et al., 2021). Alternatively, it could suggest that preclinical models do not reproduce the dopaminergic activity seen in the human condition. Another possibility is that the higher striatal dopamine release is linked to psychotic symptoms, as has been reported in other disorders that may present with psychosis (Jauhar et al., 2017; McCutcheon et al., 2020). Further studies are thus needed to test our findings particularly in patients who are resistant to serotonergic drugs and with psychotic symptoms. Notwithstanding this, it is not clear what might underlie greater striatal dopamine release in MDD, although, when taken with our finding of lower DAT availability, it could reflect a lower capacity to remove dopamine from the synapse in patients. Alternatively, given the replicated finding of lower striatal D_1_ receptor availability, one could speculate that it is an adaptive response to lower signalling through the D_1_ pathway (Hare et al., 2019). Whilst this interpretation is speculative, it is consistent with theories that impairments in dopaminergic signalling contribute to the pathophysiology of MDD (Belujon and Grace, 2017). Thus, it would be useful if future studies combined measures of dopamine release, DAT and D_1_ availability, albeit this is technically challenging.

## Conclusions

Our meta-analysis shows that striatal DAT but not receptor availability is lower in MDD compared to controls as measured using PET imaging. Subgroup analysis of dopamine release studies indicated significantly greater striatal dopamine release capacity in MDD, and there is replicated evidence that striatal D_1_ receptor availability is lower in patients with MDD. No difference was noted in measures of dopamine synthesis capacity and extra-striatal dopamine receptor availability. Further studies are needed in sub-groups such as patients with treatment-resistant depression or psychosis to further investigate the nature of the dopaminergic system in MDD.

## Supplemental Material

sj-docx-1-jop-10.1177_02698811231200881 – Supplemental material for Dopamine in major depressive disorder: A systematic review and meta-analysis of in vivo imaging studies Supplemental material, sj-docx-1-jop-10.1177_02698811231200881 for Dopamine in major depressive disorder: A systematic review and meta-analysis of in vivo imaging studies by Yuya Mizuno, Abhishekh Hulegar Ashok, Bhagyashree Bhaskar Bhat, Sameer Jauhar and Oliver D Howes in Journal of Psychopharmacology
