# Supplementary material for: Dopamine in major depressive disorder: A systematic review and meta-analysis of in vivo imaging studies
Source: J Psychopharmacol. 2023 Oct 9;37(11):1058–69. doi: 10.1177/02698811231200881 (PMC10647912; doi:10.1177/02698811231200881)
Supplement: sj-docx-1-jop-10.1177_02698811231200881 – Supplemental material for Dopamine in major depressive disorder: A systematic review and meta-analysis of in vivo imaging studies [file sj-docx-1-jop-10.1177_02698811231200881.docx]

**Supplementary Material**

Dopamine in Major Depressive Disorder: A Systematic Review and Meta-analysis of In Vivo Imaging Studies

**Supplementary Figure 1**. PRISMA flowchart showing the inclusion of studies for the meta-analysis on dopaminergic function in major depressive disorder

**Supplementary Figure 2**. Funnel plot for standardised mean difference of dopamine D_2/3_ receptor studies

**Supplementary Figure 3**. Meta-regression regarding standardised mean difference of dopamine D_2/3_ receptor studies

**Supplementary Figure 4**. Funnel plot for standardised mean difference of dopamine transporter studies

**Supplementary Figure 5**. Meta-regression regarding standardised mean difference of dopamine transporter studies

**Supplementary Figure 6**. Post-hoc analysis: Forest plot showing effect sizes for D_2/3_ receptor availability in MDD patients currently not on serotonin reuptake inhibitors

**Supplementary Table 1**. PRISMA 2020 checklist

**Supplementary Table 2**. Molecular imaging studies on D_2/3_ availability in patients with major depressive disorder compared to healthy controls

**Supplementary Table 3**. Molecular imaging studies on dopamine transporter availability in patients with major depressive disorder compared to healthy controls

**Supplementary Table 4**. Molecular imaging studies on dopamine synthesis capacity in patients with major depressive disorder compared to healthy controls

**Supplementary Table 5**. Molecular imaging studies on dopamine release in patients with major depressive disorder compared to healthy controls

**Supplementary Table 6**. Molecular imaging studies on dopamine D_1_ availability in patients with major depressive disorder compared to healthy controls

**Supplementary Table 7**. Molecular imaging studies on extra-striatal dopamine receptor availability in patients with major depressive disorder compared to healthy controls

**Supplementary Table 8**. Risk of bias assessment of included studies using the New Castle-Ottawa Scale for case-control studies

**Supplementary Table 9**. Pre-planned subgroup analyses for studies of D_2/3_ receptor availability and dopamine transporter availability

**Supplementary Figure 1.** PRISMA flowchart showing the inclusion of studies for the meta-analysis on dopaminergic function in major depressive disorder

Total records identified in electronic search (n=2,284)

(Embase, MEDLINE, and PsycINFO combined in Ovid, n=2,117;

Cochrane CENTRAL, n=88; ClinicalTrials.gov, n=79)

## **Screening**

## **Included**

## **Eligibility**

## **Identification**

Records after duplicates removed (n=1,672)

Titles and record types screened (n=1,672)

Records excluded (n=1,558)

Full-text assessed for eligibility (n=69)

Records excluded for the following reasons:

No healthy control group (n=8)

No extractable data (n=7)

Overlap with included study (n=6)

Diagnosis of bipolar disorder (n=2)

Mixed diagnoses with ≦5 patients with MDD (n=2)

Diagnosis of seasonal affective disorder (n=1)

Comorbid fibromyalgia (n=1)

Only healthy participants (n=1)

Studies included in the qualitative synthesis (n=43)

Full-text articles remaining (n=41)

Additional publications identified by hand-search

of reference lists (n=1)

Additional data provided upon request (n=1) ^*1^

Records excluded (n=45)

Abstracts screened (n=114)

Extra-striatal dopamine (n=4) ^*2^

D_1_ receptor (n=2)

Studies included in the meta-analysis (n=38)

Dopamine D_2/3_ receptor (n=20) ^*2, 3, 4^

Dopamine transporter (n=17) ^*3^

Dopamine synthesis (n=3) ^*4^

Dopamine release (n=3) ^*5^

^*1^ Additional data was provided upon request for Peciña et al. 2017; ^*2^ One study by Montgomery et al. 2007 reported on both dopamine D_2/3_ receptor and extra-striatal dopamine, ^*3^ One study by Yang et al. 2008 reported on both dopamine D_2/3_ receptor and dopamine transporter, ^*4^ One study by Wing et al. 2015 reported on both dopamine D_2/3_ receptor and dopamine synthesis, ^*5^ Studies of dopamine release were also included in the meta-analysis of dopamine D_2/3_ receptor

**Supplementary Figure 2**. Funnel plot for standardised mean difference of dopamine D_2/3_ receptor studies. Visual inspection of the funnel plot, as well as a regression test (z=0.037, *p*=0.971), did not indicate a pattern of missing negative studies.

**
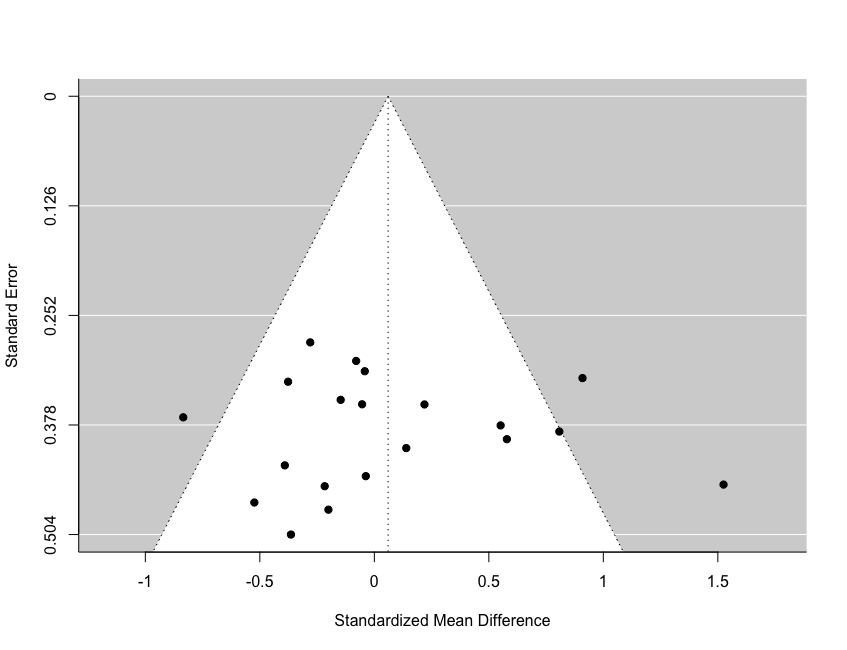
**

**Supplementary Figure 3**. Meta-regression regarding standardised mean differences of dopamine D_2/3_ receptor studies

S3a. Meta-regression of dopamine D_2/3_ receptors against mean age of patients in included studies. The results show that mean age is not a significant moderator of SMD for dopamine D_2/3_ receptors, z=-1.211; p=0.226.


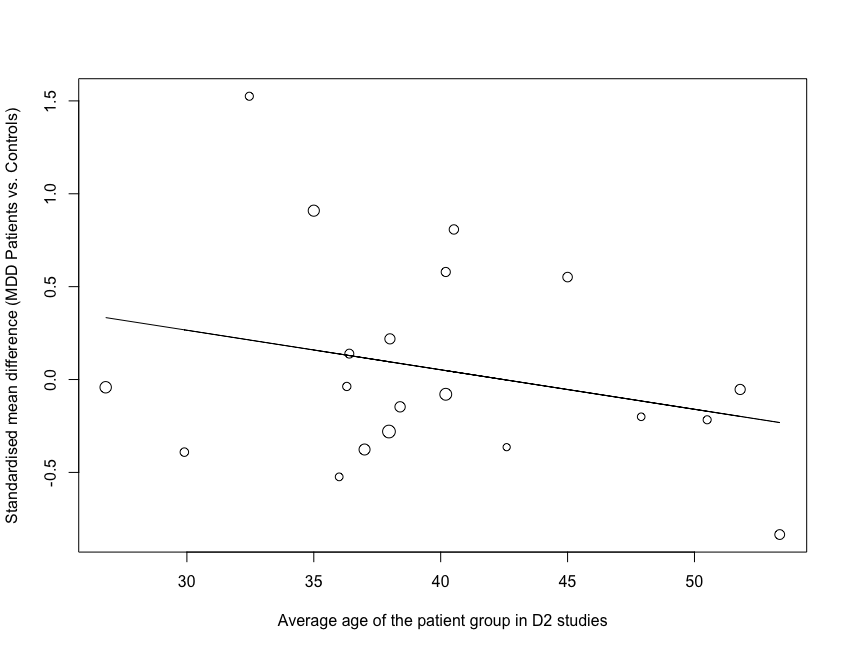


S3b. Meta-regression of dopamine D_2/3_ receptors against total HAM-D 17 equivalent depression severity of patients in included studies. The results show that depression severity is not a significant moderator of SMD for dopamine D_2/3_ receptors, z=0.310; p=0.756.


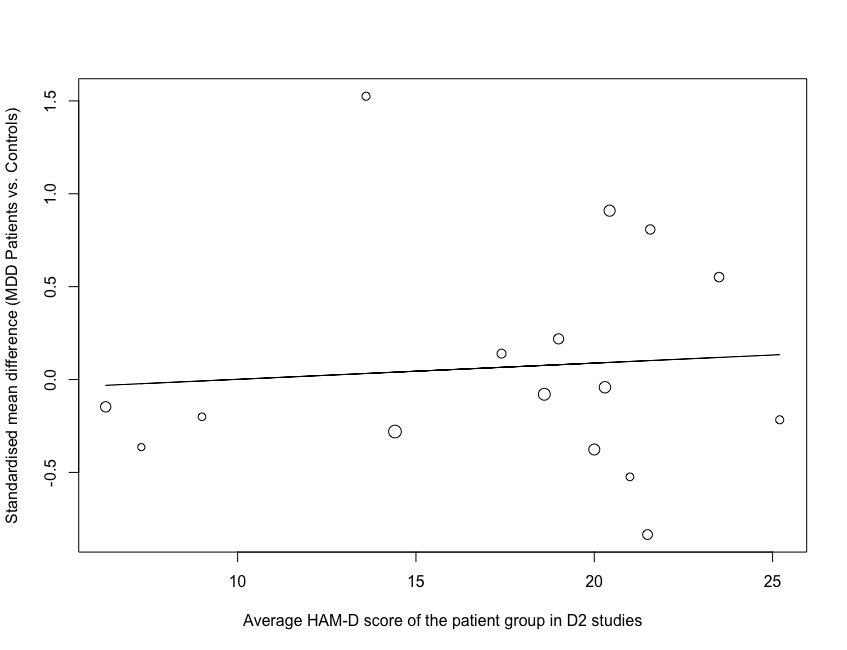


**Supplementary Figure 4**. Funnel plot for standardised mean difference of dopamine transporter studies. Visual inspection of the funnel plot and regression test (z=-3.476, *p*<0.001) indicates the possibility of publication bias, with small studies with large postive effect sizes possibly unpublished. However, the asymmetry in the funnel plot is largely due to the study by Wu et al. 2011, which reported a large negative effect size of 4.50.


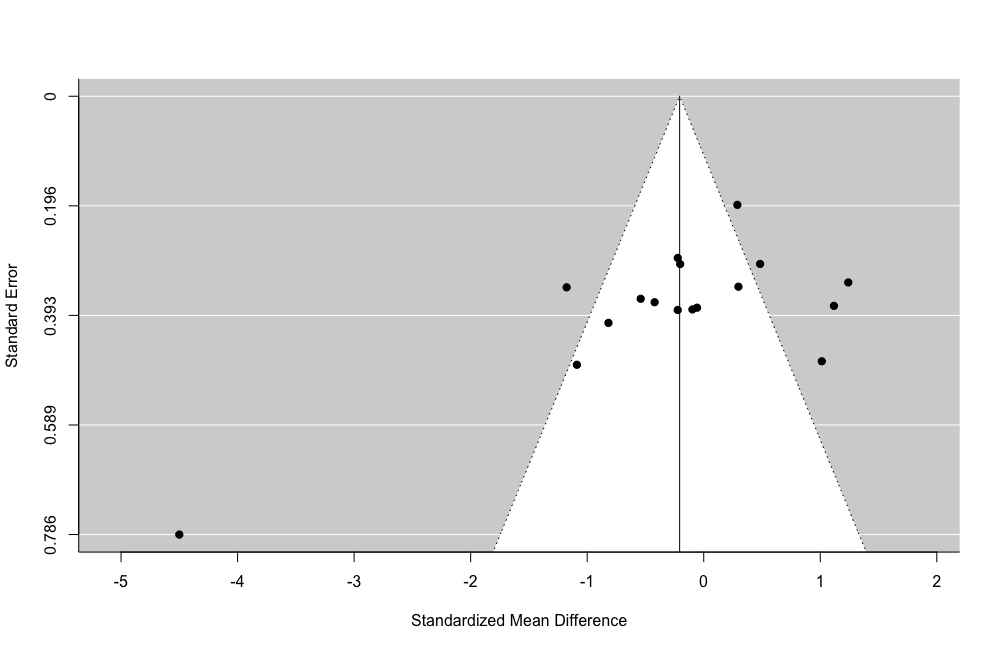


**Supplementary Figure 5**. Meta-regression regarding standardised mean differences of dopamine transporter studies

S5a. Meta-regression of dopamine transporters against mean age of patients in included studies. The results show that mean age is not a significant moderator of SMD for dopamine transporters, z=-1.103; p=0.270.


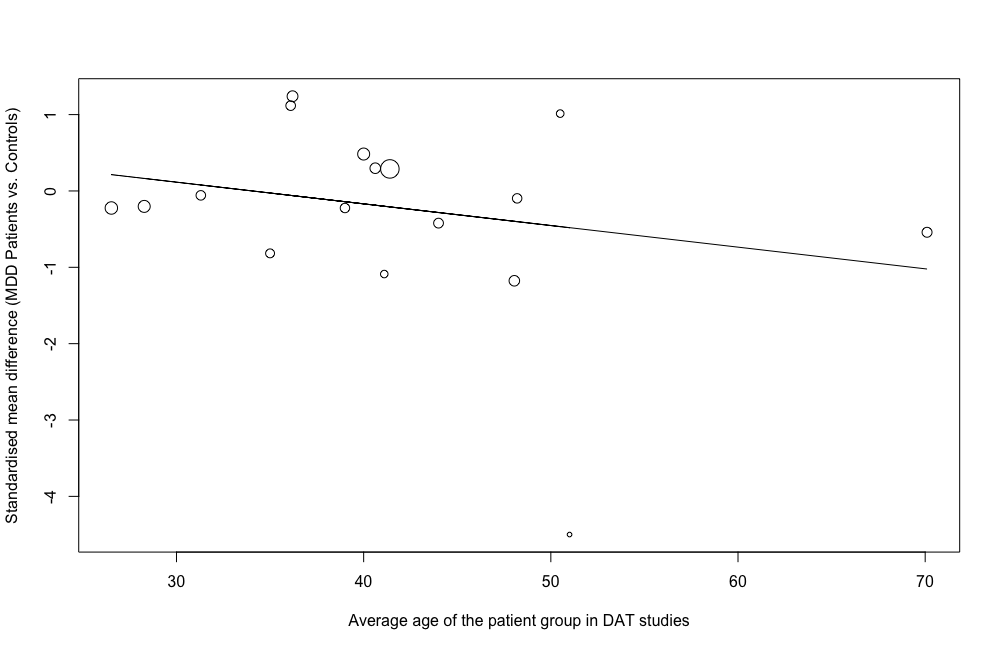


S5b. Meta-regression of dopamine transporters against total HAM-D 17 equivalent depression severity of patients in included studies. The results show that depression severity is not a significant moderator of SMD for dopamine transporters, z=-0.052; p=0.959.


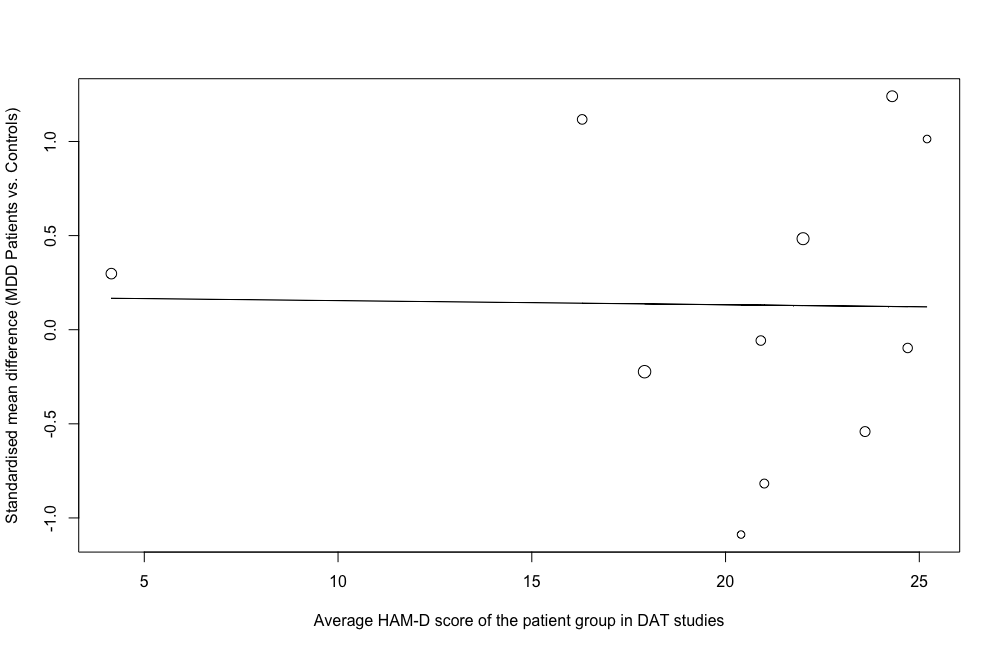


**Supplementary Figure 6**. Post-hoc analysis: Forest plot showing effect sizes for D_2/3_ receptor availability in MDD patients currently not on serotonin reuptake inhibitors

The forest plot shows effect sizes using a random-effects model, with 95% Confidence Intervals for striatal dopamine D_2/3_ receptor availability. Dopamine D_2/3_ receptor availability did not differ significantly between people with MDD who were currently not on serotonin reuptake inhibitors and healthy controls (Hedges’ g=-0.15 [95% CI, -0.53 – 0.22], p=0.428).


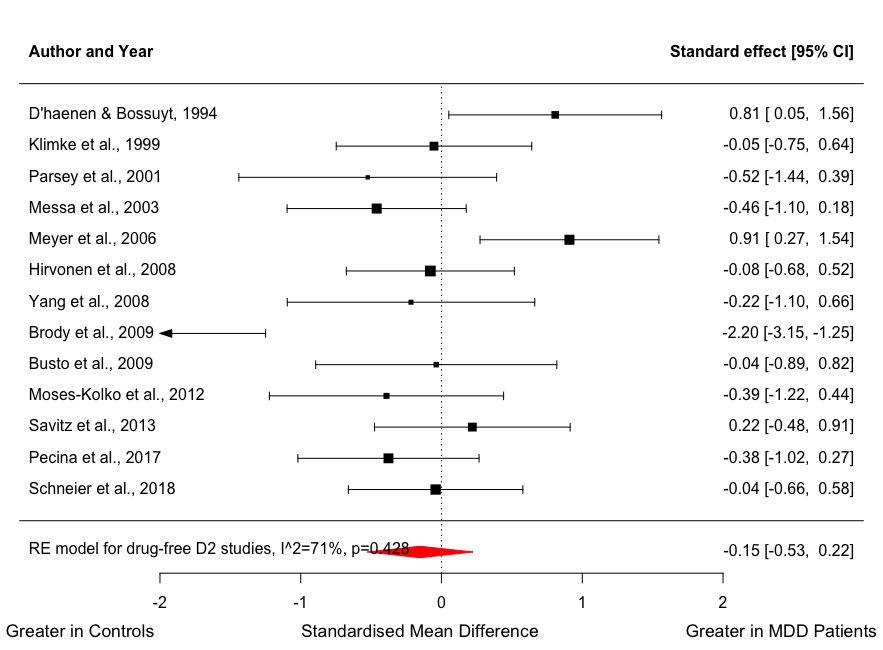


**Supplementary Table 1**. PRISMA 2020 checklist

| **Section and Topic** | **Item #** | **Checklist item** | **Location where item is reported** |
| --- | --- | --- | --- |
| **TITLE** | | |  |
| Title | 1 | Identify the report as a systematic review. | Page 1 |
| **ABSTRACT** | | |  |
| Abstract | 2 | See the PRISMA 2020 for Abstracts checklist. | Pages 1-2 |
| **INTRODUCTION** | | |  |
| Rationale | 3 | Describe the rationale for the review in the context of existing knowledge. | Pages 2-3 |
| Objectives | 4 | Provide an explicit statement of the objective(s) or question(s) the review addresses. | Page 3 |
| **METHODS** | | |  |
| Eligibility criteria | 5 | Specify the inclusion and exclusion criteria for the review and how studies were grouped for the syntheses. | Page 4-5 |
| Information sources | 6 | Specify all databases, registers, websites, organisations, reference lists and other sources searched or consulted to identify studies. Specify the date when each source was last searched or consulted. | Page 4 |
| Search strategy | 7 | Present the full search strategies for all databases, registers and websites, including any filters and limits used. | Page 4 |
| Selection process | 8 | Specify the methods used to decide whether a study met the inclusion criteria of the review, including how many reviewers screened each record and each report retrieved, whether they worked independently, and if applicable, details of automation tools used in the process. | Page 5 |
| Data collection process | 9 | Specify the methods used to collect data from reports, including how many reviewers collected data from each report, whether they worked independently, any processes for obtaining or confirming data from study investigators, and if applicable, details of automation tools used in the process. | Pages 5-6 |
| Data items | 10a | List and define all outcomes for which data were sought. Specify whether all results that were compatible with each outcome domain in each study were sought (e.g. for all measures, time points, analyses), and if not, the methods used to decide which results to collect. | Pages 6-7 |
|  | 10b | List and define all other variables for which data were sought (e.g. participant and intervention characteristics, funding sources). Describe any assumptions made about any missing or unclear information. | Page 5 |
| Study risk of bias assessment | 11 | Specify the methods used to assess risk of bias in the included studies, including details of the tool(s) used, how many reviewers assessed each study and whether they worked independently, and if applicable, details of automation tools used in the process. | Page 6 |
| Effect measures | 12 | Specify for each outcome the effect measure(s) (e.g. risk ratio, mean difference) used in the synthesis or presentation of results. | Page 7 |

| Synthesis methods | 13a | Describe the processes used to decide which studies were eligible for each synthesis (e.g. tabulating the study intervention characteristics and comparing against the planned groups for each synthesis (item #5)). | Pages 5-7 |
| --- | --- | --- | --- |
|  | 13b | Describe any methods required to prepare the data for presentation or synthesis, such as handling of missing summary statistics, or data conversions. | Page 7 |
|  | 13c | Describe any methods used to tabulate or visually display results of individual studies and syntheses. | Page 7 |
|  | 13d | Describe any methods used to synthesize results and provide a rationale for the choice(s). If meta-analysis was performed, describe the model(s), method(s) to identify the presence and extent of statistical heterogeneity, and software package(s) used. | Page 7 |
|  | 13e | Describe any methods used to explore possible causes of heterogeneity among study results (e.g. subgroup analysis, meta-regression). | Page 7-8 |
|  | 13f | Describe any sensitivity analyses conducted to assess robustness of the synthesized results. | Page 7-8 |
| Reporting bias assessment | 14 | Describe any methods used to assess risk of bias due to missing results in a synthesis (arising from reporting biases). | Page 8 |
| Certainty assessment | 15 | Describe any methods used to assess certainty (or confidence) in the body of evidence for an outcome. | Page 7-8 |
| **RESULTS** | | |  |
| Study selection | 16a | Describe the results of the search and selection process, from the number of records identified in the search to the number of studies included in the review, ideally using a flow diagram. | Page 9, Supplementary Figure 1 |
|  | 16b | Cite studies that might appear to meet the inclusion criteria, but which were excluded, and explain why they were excluded. | Supplementary Figure 1 |
| Study characteristics | 17 | Cite each included study and present its characteristics. | Page 9-14, Supplementary Tables 2-7 |
| Risk of bias in studies | 18 | Present assessments of risk of bias for each included study. | Page 9, Supplementary Table 8 |
| Results of individual studies | 19 | For all outcomes, present, for each study: (a) summary statistics for each group (where appropriate) and (b) an effect estimate and its precision (e.g. confidence/credible interval), ideally using structured tables or plots. | Page 9-14 |
| Results of syntheses | 20a | For each synthesis, briefly summarise the characteristics and risk of bias among contributing studies. | Page 9-14 |
|  | 20b | Present results of all statistical syntheses conducted. If meta-analysis was done, present for each the summary estimate and its precision (e.g. confidence/credible interval) and measures of statistical heterogeneity. If comparing groups, describe the direction of the effect. | Page 9-13 |
|  | 20c | Present results of all investigations of possible causes of heterogeneity among study results. | Page 9-13 |
|  | 20d | Present results of all sensitivity analyses conducted to assess the robustness of the synthesized results. | Page 9-13 |
| Reporting biases | 21 | Present assessments of risk of bias due to missing results (arising from reporting biases) for each synthesis assessed. | Page 9-11, Supplementary Figures 2 & 4 |
| Certainty of evidence | 22 | Present assessments of certainty (or confidence) in the body of evidence for each outcome assessed. | Page 11-12 |
| **DISCUSSION** | | |  |
| Discussion | 23a | Provide a general interpretation of the results in the context of other evidence. | Page 14-15 |
|  | 23b | Discuss any limitations of the evidence included in the review. | Page 16-18 |
|  | 23c | Discuss any limitations of the review processes used. | Page 16-17 |
|  | 23d | Discuss implications of the results for practice, policy, and future research. | Page 18-19 |
| **OTHER INFORMATION** | | |  |
| Registration and protocol | 24a | Provide registration information for the review, including register name and registration number, or state that the review was not registered. | Page 4 |
|  | 24b | Indicate where the review protocol can be accessed, or state that a protocol was not prepared. | Page 4 |
|  | 24c | Describe and explain any amendments to information provided at registration or in the protocol. | N/A |
| Support | 25 | Describe sources of financial or non-financial support for the review, and the role of the funders or sponsors in the review. | Page 20 |
| Competing interests | 26 | Declare any competing interests of review authors. | Page 20 |
| Availability of data, code and other materials | 27 | Report which of the following are publicly available and where they can be found: template data collection forms; data extracted from included studies; data used for all analyses; analytic code; any other materials used in the review. | Page 21 |

*From:*  Page MJ, McKenzie JE, Bossuyt PM, Boutron I, Hoffmann TC, Mulrow CD, et al. The PRISMA 2020 statement: an updated guideline for reporting systematic reviews. BMJ 2021;372:n71. doi: 10.1136/bmj.n71

**Supplementary Table 2**. Molecular imaging studies on D_2/3_ availability in patients with major depressive disorder compared to healthy controls

| **Author/year** | **Patients/**  **Controls, n** | **Diagnosis** | **Psychotropic treatment** | **Region of interest** | **Reference region** | **Tracer** | **Measure** | **Results in patients compared to controls** |
| --- | --- | --- | --- | --- | --- | --- | --- | --- |
| D’haenen & Bossuyt 1994 | 21/11 | DSM-III-R, major depression | Underwent psychotropic washout period of ≧7 days except for four patients receiving a hypnotic, four patients previously treated with low doses of a neuroleptic | Basal ganglia | Cerebellum | [^123^I]IBZM | Basal ganglia /cerebellum uptake ratio | ↑ |
| Ebert et al. 1996 | 20/10 | DSM-III-R, major depressive episode | Ten patients had received amitriptyline 150mg/day for ≧2 weeks, ten patients were free of psychoactive drugs for ≧6 months, all were drug-naïve for neuroleptic medication | Basal ganglia | Cerebellum | [^123^I]IBZM | Basal ganglia /cerebellum uptake ratio | ↔ |
| Shah et al. 1997 | 14/15 | DSM-III-R, major depressive episode, two with bipolar affective disorder | Eight patients on antidepressant medication (including lithium) only, three patients on benzodiazepines only, four patients were free of all medications, all had not received neuroleptics for ≧3 months | Striatum | Whole slice | [^123^I]IBZM | Striatum/whole slice uptake ratio | ↑ in right striatum only |
| Klimke et al. 1999 | 15/17 | DSM-IV major depression | All patients were non-responsive to pre-treatment with a standard tricyclic antidepressant, underwent a 1-week washout period with exception to benzodiazepines, no treatment with dopaminergic drugs for ≧6 months | Striatum | Cerebellum | [^123^I]IBZM | Striatum /cerebellum ratio | ↔ |
| Parsey et al. 2001 | 9/10 | DSM-IV unipolar major depression, four patients also met an Axis II diagnosis | Four patients had never received any antidepressants, four patients had been treated once before with antidepressants, and one patient had two previously treated episodes, none had been treated with antidepressants for ≧6 months | Striatum | Frontal and occipital regions | [^123^I]IBZM | Striatal equilibrium specific to nonspecific partition coefficient (V_3_’’) | ↔ |
| Messa et al. 2003 | 34/20 | DSM-IV single or recurrent major depressive episode | 19 patients never treated with antidepressant drugs, mood stabilizers or neuroleptics, 15 patients in their 4^th^ week of treatment with paroxetine, benzodiazepines were allowed | Striatum | Cerebellum | [^18^F]fluoroethylspiperone | Binding index | ↔ |
| Kuroda et al. 2006 | 8/16 | DSM-IV major depressive disorder | Patients were resistant to or intolerant to at least 2 antidepressant treatments, three patients had taken atypical antipsychotics as augmentation, all had washout period of ≧4 weeks, however fluvoxamine and lorazepam were allowed during washout | Striatum | Cerebellum | [^11^C]raclopride | Binding potential | ↔ |
| Meyer et al. 2006 | 21/21 | DSM-IV major depressive episode secondary to major depressive disorder | 12 patients had no history of antidepressant treatment, 4 patients had this within the previous year but not previous 6-months, and 5 patients had this but not within the previous year | Striatum | Cerebellum | [^11^C]raclopride | Binding potential | ↑ |
| Montgomery et al. 2007 | 8/8 | DSM-IV major depressive disorder | All patients had regular use of an SSRI antidepressant for more than 2 months and were currently recovering | Striatum | Cerebellum | [^11^C]raclopride | Binding potential | ↓ in dorsal striatum only |
| Hirvonen et al. 2008 | 25/19 | Mild to moderate episode of DSM-IV major depressive disorder | 24 patients were antidepressant naïve, one patient had been treated with citalopram 5-years prior to imaging and had been free of antidepressants since | Striatum | Cerebellum | [^11^C]raclopride | BP_ND_ | ↔ |
| Yang et al. 2008 | 10/10 | DSM-IV major depressive disorder | None of the patients had been treated with antidepressants in the 3 months preceding imaging | Striatum | Occipital cortex | [^123^I]IBZM | Striatum /occipital cortex ratio | ↔ |
| Brody et al. 2009 | 10/46 | History of DSM-IV major depressive disorder | Four subjects were taking antidepressants at a stable dose for over 2 months | Striatum | Cerebellum | [^11^C]raclopride | Binding potential | ↔ |
| Busto et al. 2009 | 10/11 | DSM-IV major depressive disorder | No patients were currently taking psychotropic medications | Striatum | Cerebellum | [^11^C]raclopride | Binding potential | ↔ |
| Moses-Kolko et al. 2012 | 10/13 | DSM-IV major depressive disorder, unipolar depression | Eight patients were antidepressant naïve, no patients had prior antipsychotic exposure, all patients were free of psychotropic drug exposure for a minimum of 3 weeks before scanning | Striatum | Cerebellum | [^11^C]raclopride | BP_ND_ | ↓ |
| Savitz et al. 2013 | 12/24 | DSM-IV-TR major depressive disorder, current depressive episode | No patients were taking psychotropic medications within 3 weeks of scanning (8 weeks for fluoxetine) | Striatum | Cerebellum | [^11^C]raclopride | BP_ND_ | ↔ |
| De Kwaasteniet et al. 2014 | 17/15 | DSM-IV major depressive disorder meeting study criteria for treatment resistant depression | 11 patients were taking antipsychotics, three patients were not receiving any psychotropic medications | Striatum | Occipital cortex | [^123^I]IBZM | BP_ND_ | ↔ |
| Wing et al. 2015 | 8/10 | DSM-IV major depressive disorder | All patients were receiving treatment with antidepressants | Striatum | Cerebellum | [^11^C]raclopride | Standardized uptake value (SUV) | ↔ |
| Peciña et al. 2017 | 23/16 | DSM-5 moderate-severe major depressive disorder | None of the patients were taking any psychotropic medications at the time of the study for at least 6-months, except for occasional use of sleep aids (frequency less than twice per week, and none for at least 1-week prior to the study) | Striatum | Cerebellum | [^11^C]raclopride | BP_ND_ | ↑ in bilateral ventral pallidum/nucleus accumbens, and the right ventral caudate and putamen  (Additional data regarding whole striatum provided upon request) |
| Hamilton et al. 2018 | 16/14 | DSM-5 major depressive disorder | 2 patients were receiving bupropion, other patients were not taking any psychoactive medications | Striatum | Cerebellum | [^11^C]raclopride | BP_ND_ | ↑ in the ventral striatum bilaterally, and in the right dorsal striatum |
| Schneier et al. 2018 | 20/20 | DSM-IV major depressive disorder | Treatment-naïve (<2 weeks of lifetime psychiatric medication and none for the past 3-months) | Globus pallidus, precommissural dorsal caudate, postcommissural caudate, precommissural dorsal putamen, postcommissural putamen, ventral striatum, midbrain, and thalamus | Cerebellum | [^11^C](+)PHNO | BP_ND_ | ↔ |

**Supplementary Table 3**. Molecular imaging studies on dopamine transporter availability in patients with major depressive disorder compared to healthy controls

| **Author/year** | **Patients/**  **Controls, n** | **Diagnosis** | **Psychotropic treatment** | **Region of interest** | **Reference region** | **Tracer** | **Measure** | **Results in patients compared to controls** |
| --- | --- | --- | --- | --- | --- | --- | --- | --- |
| Malison et al. 1998 | 15/15 | DSM-III-R unipolar major depressive disorder | Free of antidepressants and non-psychotropic medication for a minimum of 3 weeks | Striatum and brainstem | Occipital cortex | [^123^I]β-CIT | V_3_’’ | ↔ |
| Laasonen-Balk et al. 1999 | 15/18 | DSM-III-R, major depression | No antidepressant treatment prior to SPECT imaging (drug-naïve) | Striatum | White matter | [^123^I]β-CIT | Striatum-to-white matter ratio | ↑ |
| Meyer et al. 2001 | 9/23 | DSM-IV, major depressive episode secondary to MDD | Free of psychotropic drugs for at least 3-months, only 2 patients had taken antidepressant medication within the past year, 5 patients were treatment naive | Left and right caudate and putamen, bilateral striatum | Cerebellum | [^11^C]RTI-32 | Binding potential (BP) | ↓ |
| Brunswick et al. 2003 | 15/46 | DSM-IV major depressive episode | No antidepressants for at least 7-days, no MAOIs for at least 2-weeks, and no fluoxetine for at least 3-weeks | Right and left putamen (anterior, posterior) and caudate | None | [^99m^Tc]TRODAT-1 | Specific uptake (k3/k4) | ↑ in right anterior and posterior putamen, left posterior putamen, and left caudate |
| Argyelán et al. 2005 | 16/12 | DSM-IV depression | Venlafaxine, sertraline or antipsychotic medication were stopped 6-months before, 2-week wash-out period for other antidepressants, none had previously received fluoxetine, 7 patients were drug naive | Striatum | Occipital cortex | [^99m^Tc]TRODAT-1 | Binding potential | ↔ |
| Lehto et al. 2006 | 29/18 | DSM-IV depression (melancholic, non-differentiated, and atypical subtypes) | Drug naive | Striatum | Cerebellum | [^123^I]nor-β-CIT | Distribution volume ratio | ↔ |
| Sarchiapone et al. 2006 | 11/9 | DSM-IV-TR major depressive episode with anhedonia (9 patients with MDD, 2 patients with bipolar II disorder) | All patients were drug-free at the time of scan for at least 4 weeks. Six patients were drug-naïve. | Striatum | Occipital cortex | [^123^I]β-CIT | Striatum-to-occipital cortex ratio | ↓ |
| Lehto et al. 2008 | 11/19 | DSM-IV severe or moderate major depression | Drug-naïve | Striatum | Cerebellum | [^123^I]nor-β-CIT | Distribution volume ratio | ↔ |
| Yang et al. 2008 | 10/10 | DSM-IV major depression | All patients were free of antidepressant medication in the 3 months preceding the study. | Striatum | Occipital cortex | [^99m^Tc]TRODAT-1 | Striatum-to-occipital cortex ratio | ↑ |
| Hsieh et al. 2010 | 13/26 | Euthymic patients with a history of DSM-IV major depressive disorder | All patients were drug-free for more than 3 months. | Striatum | Occipital cortex | [^99m^Tc]TRODAT-1 | Striatum-to-occipital cortex ratio | ↔ |
| Wu et al. 2011 | 13/10 | DSM-IV major depressive episode | None of the patients had received dopaminergic drugs, antidepressants, or antipsychotic drugs within the previous 2 years. | Right and left striatum | Cerebellum | [^99m^Tc]TRODAT-1 | Striatum-to-cerebellum ratio | ↓ |
| Amsterdam et al. 2012 | 39/84 | DSM-IV major depressive episode, 24 with unipolar depression, 15 with bipolar II depression | None of the patients had used psychotropic medication within the preceding 6 months | Right and left putamen and caudate | Supratentorial ROI | [^99m^Tc]TRODAT-1 | Distribution volume ratio | ↑ in right and left putamen only |
| Hsiao et al. 2013 | 23/20 | DSM-IV first episode major depressive disorder | Free of medication for at least 6 months | Right and left caudate and putamen | Occipital cortex | [^99m^Tc]TRODAT-1 | Specific update ratio | ↑ |
| Camardese et al. 2014 | 20/20 | DSM-IV-TR major depressive disorder | Free of psychotropic drugs for at least 4-weeks before scan | Right and left striatum, caudate and putamen | Occipital cortex | [^123^I]FP-CIT | Specific to non-specific binding ratios | ↓ |
| Hellwig et al. 2018 | 16/12 | Inpatients with DSM-IV major depressive disorder, referred for ECT due to insufficient response to at least two first-line antidepressants | Free of antidepressants, neuroleptics, and psychostimulants for at least 5× plasma half-life (at least 7 days) | Bilateral striatum | Occipital cortex | [^123^I]FP-CIT | BP_ND_ | ↔ |
| Pizzagalli et al. 2019 | 25/23 | DSM-IV major depressive episode | Free of any psychotropic medication in the past 2 weeks (6 week for fluoxetine, 6 months for dopaminergic drugs) | Bilateral striatum (caudate, putamen, accumbens) and midbrain (VTA) | Cerebellum | [^11^C]altropane | BP_ND_ | ↓ in putamen and VTA only |
| Moriya et al. 2020 | 11/27 | DSM-IV major depressive disorder | All patients were receiving treatment with antidepressants | Bilateral caudate, putamen, nucleus accumbens, and substantia nigra | Cerebellum | [^18^F]FE-PE2I | BP_ND_ | ↓ in nucleus accumbens only |

**Supplementary Table 4**. Molecular imaging studies on dopamine synthesis capacity in patients with major depressive disorder compared to healthy controls

| **Author/year** | **Patients/**  **Controls, n** | **Diagnosis** | **Psychotropic treatment** | **Region of interest** | **Reference region** | **Tracer** | **Measure** | **Results in patients compared to controls** |
| --- | --- | --- | --- | --- | --- | --- | --- | --- |
| Agren et al. 1993 | 7/11 | DSM-III-R current major depression | Drug-free for at least 14 days, 2 patients were drug-naïve | Bilateral caudate nucleus and lentiform nucleus | Whole brain | [^11^C]L-DOPA | k_3_ | ↔ |
| Martinot et al. 2001 | 12/10 | DSM-IV major depressive disorder | 6 patients were taking SSRIs, 6 patients were drug-free | Left and right caudate and putamen | Occipital cortex | [^18^F]DOPA | K_i_ | ↓ only in the left caudate of patients with psychomotor retardation |
| Wing et al. 2015 | 8/10 | DSM-IV major depressive disorder | All patients were receiving treatment with antidepressants | Left and right putamen and caudate | Cerebellum | [^18^F]DOPA | K_i_ | ↔ |

**Supplementary Table 5**. Molecular imaging studies on dopamine release in patients with major depressive disorder compared to healthy controls

| **Author/year** | **Patients/**  **Controls, n** | **Dopamine release paradigm** | **Diagnosis** | **Psychotropic treatment** | **Region of interest** | **Reference region** | **Tracer** | **Measure** | **Results in patients compared to controls** |
| --- | --- | --- | --- | --- | --- | --- | --- | --- | --- |
| Parsey et al. 2001 | 9/10 | i.v. d-amphetamine challenge (0.3 mg/kg) | DSM-IV unipolar major depression, four patients also met an Axis II diagnosis | Four patients had never received any antidepressants, four patients had been treated once before with antidepressants, and one patient had two previously treated episodes, none had been treated with antidepressants for ≧6 months | Striatum | Frontal and occipital regions | [^123^I]IBZM | Striatal equilibrium specific to nonspecific partition coefficient (V_3_’’) | ↔ |
| Busto et al. 2009 | 10/11 | Oral d-amphetamine sulfate 30mg | DSM-IV major depressive disorder | No patients were currently taking psychotropic medications | Striatum | Cerebellum | [^11^C]raclopride | Binding potential | ↔ |
| Schneier et al. 2018 | 20/20 | Oral amphetamine (0.5 mg/kg) | DSM-IV major depressive disorder | Treatment-naïve (<2 weeks of lifetime psychiatric medication and none for the past 3-months) | Globus pallidus, precommissural dorsal caudate, postcommissural caudate, precommissural dorsal putamen, postcommissural putamen, ventral striatum, midbrain, and thalamus | Cerebellum | [^11^C](+)PHNO | BP_ND_ | ↔ |

**Supplementary Table 6**. Molecular imaging studies on dopamine D_1_ availability in patients with major depressive disorder compared to healthy controls

| **Author/year** | **Patients/**  **Controls, n** | **Diagnosis** | **Psychotropic treatment** | **Region of interest** | **Reference region** | **Tracer** | **Measure** | **Results in patients compared to controls** |
| --- | --- | --- | --- | --- | --- | --- | --- | --- |
| Dougherty et al. 2006 | 10/10 | DSM-IV major depressive disorder, single episode or recurrent | No use of psychotropic medication within 21 days (40 days for fluoxetine) | Striatum | Cerebellum | [^11^C]SCH 23390 | Binding potential | ↓ |
| Cannon et al. 2009 | 18/19 | DSM-IV recurrent or chronic major depressive disorder | 11 patients were treatment-naïve, all patients were free of psychotropic drugs within the 3-weeks before scanning (8-weeks for fluoxetine) | Anteroventral striatum, dorsal caudate, middle caudate, ventral putamen, and dorsal putamen | Cerebellar cortex | [^11^C]NNC-112 | BP_ND_ | ↓ only in the left middle caudate |

**Supplementary Table 7**. Molecular imaging studies on extra-striatal dopamine receptor availability in patients with major depressive disorder compared to healthy controls

| **Author/year** | **Patients/**  **Controls, n** | **Diagnosis** | **Psychotropic treatment** | **Region of interest** | **Reference region** | **Tracer** | **Measure** | **Results in patients compared to controls** |
| --- | --- | --- | --- | --- | --- | --- | --- | --- |
| Montgomery et al. 2007 | 7/7 | DSM-IV major depressive disorder | Absent of any psychotropic medication use for at least 3-months prior to the scan | Bilateral caudate nucleus and lentiform nucleus | Amygdala, hippocampus, frontal cortex, anterior cingulate gyrus, thalamus, brain stem, cerebellum | [^11^C]FLB 457 | Volume of distribution | ↔ |
| Lehto et al. 2008 | 10/10 | DSM-IV major depressive disorder (6 patients current, 4 patients in partial remission) | No patients had ever used antipsychotics, no patients used antidepressants 6-months prior or benzodiazepines 3-months prior | Temporal cortex | Cerebellum | [^123^I]Epidepride | Specific binding | ↔ |
| Saijo et al. 2010 | 7/11 | DSM-IV major depressive disorder | All patients were receiving treatment with SSRIs (paroxetine or fluvoxamine) | Voxel-based method | Cerebellum | [^11^C]FLB 457 | Binding potential | ↔ |
| Dubol et al. 2020 | 8/24 | DSM-IV-TR major depressive disorder | All patients were receiving antidepressant treatment in monotherapy, low doses of benzodiazepines were allowed | Voxel-based method | Cerebellum | [^11^C]PE2I | BP_ND_ | ↓ only in the superior part of the midbrain including the substantia nigra and the ventral tegmental area |

**Supplementary Table 8**. Risk of bias assessment of included studies using the New Castle-Ottawa Scale for case-control studies

| **Study type** | **Author/year** | **Selection** | **Comparability** | **Exposure** | **Quality Rating ^a^** |
| --- | --- | --- | --- | --- | --- |
|  |  | **1 2 3 4** | **Age Sex** | **1 2 3 -N/A** |  |
| **D_2/3_ availability studies** | D’haenen & Bossuyt 1994 | * * |  | * * | Poor |
|  | Ebert et al. 1996 | * * | * | * * | Fair |
|  | Shah et al. 1997 | * * | * * | * * | Fair |
|  | Klimke et al. 1999 | * * | * * | * * | Fair |
|  | Parsey et al. 2001 | * * | * * | * * | Fair |
|  | Messa et al. 2003 | * * | * | * * | Fair |
|  | Kuroda et al. 2006 | * * * | * | * * | Good |
|  | Meyer et al. 2006 | * * | * * | * * | Fair |
|  | Montgomery et al. 2007 | * * * * | * | * * | Good |
|  | Hirvonen et al. 2008 | * * * | * * | * * | Good |
|  | Yang et al. 2008 | * * | * | * * | Fair |
|  | Brody et al. 2009 | * * * * | * * | * * | Good |
|  | Busto et al. 2009 | * * * * | * | * * | Good |
|  | Moses-Kolko et al. 2012 | * * | * * | * * | Fair |
|  | Savitz et al. 2013 | * * | * * | * * | Fair |
|  | de Kwaasteniet et al. 2014 | * * | * * | * * | Fair |
|  | Wing et al. 2015 | * * * | * * | * * | Good |
|  | Peciña et al. 2017 | * * * | * | * * | Good |
|  | Hamilton et al. 2018 | * * | * | * * | Fair |
|  | Schneier et al. 2018 | * * * | * * | * * | Good |
| **Dopamine transporter studies** | Malison et al. 1998 | * * * | * * | * * | Good |
|  | Laasonen-Balk et al. 1999 | * * * | * * | * * | Good |
|  | Meyer et al. 2001 | * * * | * * | * * | Good |
|  | Brunswick et al. 2003 | * * * | * * | * * | Good |
|  | Argyelán et al. 2005 | * * | * | * * | Fair |
|  | Lehto et al. 2006 | * * * | * * | * * | Good |
|  | Sarchiapone et al. 2006 | * * * | * * | * * | Good |
|  | Lehto et al. 2008 | * * * | * * | * * | Good |
|  | Yang et al. 2008 | * * * | * | * * | Good |
|  | Hsieh et al. 2010 | * * * | * * | * * | Good |
|  | Wu et al. 2011 | * * |  | * * | Poor |
|  | Amsterdam et al. 2012 | * * * * | * * | * * | Good |
|  | Hsiao et al. 2013 | * * | * * | * * | Fair |
|  | Camardese et al. 2014 | * * | * | * * | Fair |
|  | Hellwig et al. 2018 | * * * * | * * | * * | Good |
|  | Pizzagalli et al. 2019 | * * | * | * * | Fair |
|  | Moriya et al. 2020 | * * | * | * * | Fair |
| **Dopamine synthesis capacity studies** | Agren et al. 1993 | * | * | * * | Poor |
|  | Martinot et al. 2001 | * * * * | * | * * | Good |
|  | Wing et al. 2015 | * * * | * * | * * | Good |
| **Dopamine release studies** | Parsey et al. 2001 | * * | * * | * * | Fair |
|  | Busto et al. 2009 | * * * * | * | * * | Good |
|  | Schneier et al. 2018 | * * * | * * | * * | Good |
| **Dopamine D_1_ studies** | Dougherty et al. 2006 | * * * | * * | * * | Good |
|  | Cannon et al. 2009 | * * | * * | * * | Fair |
| **Extra-striatal dopamine studies** | Montgomery et al. 2007 | * * * * | * | * * | Good |
|  | Lehto et al. 2008 | * * * * | * | * * | Good |
|  | Saijo et al. 2010 | * * * | * | * * | Good |
|  | Dubol et al. 2020 | * | * * | * * | Poor |

^a^ Quality ratings are based on previously reported thresholds for converting the Newcastle-Ottawa scales to AHRQ standards

**Good quality**: 3 or 4 stars in selection domain AND 1 or 2 stars in comparability domain AND 2 or 3 stars in outcome/exposure domain

**Fair quality:** 2 stars in selection domain AND 1 or 2 stars in comparability domain AND 2 or 3 stars in outcome/exposure domain

**Poor quality:** 0 or 1 star in selection domain OR 0 stars in comparability domain OR 0 or 1 stars in outcome/exposure domain

**Supplementary Table 9**. Pre-planned subgroup analyses for studies of D_2/3_ receptor availability and dopamine transporter availability

| Analysis | **SMD** | 95% CI | *I^2^* | *p*-value |
| --- | --- | --- | --- | --- |
| ***D_2/3_ receptor availability*** |  |  |  |  |
| #1 Original analysis including all studies (N=20) | 0.06 | -0.18 – 0.30 | 51% | 0.620 |
| #1.1 Radiotracer used |  |  |  |  |
| [^11^C]raclopride studies (N=11) | 0.11 | -0.23 – 0.45 | 55% | 0.516 |
| [^123^I]IBZM studies (N=7) | 0.06 | -0.41 – 0.52 | 60% | 0.813 |
| [^18^F]FESP (N=1) | NA | | | |
| [^11^C]-(+)-PHNO (N=1) | NA | | | |
| #1.2 Drug-naïve patients with MDD |  | | | |
| No (N=18) | 0.08 | -0.19 – 0.35 | 56% | 0.579 |
| Yes (N=2) | NA |  |  |  |
| #1.3 Treatment-resistant patients with MDD |  |  |  |  |
| No (N=18) | 0.11 | -0.14 – 0.35 | 48% | 0.385 |
| Yes (N=2) | NA |  |  |  |
| ***Dopamine transporter availability*** |  |  |  |  |
| #2 Original analysis including all studies (N=17) | -0.21 | -0.71 – 0.30 | 89% | 0.420 |
| #2.1 Radiotracer used |  | | | |
| [^99^mTc]TRODAT-1 studies (N=7) | -0.11 | -1.42 – 1.19 | 96% | 0.864 |
| [^123^I]B-CIT studies (N=3) | NA |  |  |  |
| [^123^I]nor-B-CIT studies (N=2) | NA |  |  |  |
| [^123^I]FP-CIT studies (N=2) | NA |  |  |  |
| [^11^C]RTI-32 studies (N=1) | NA |  |  |  |
| [^11^C]altropane studies (N=1) | NA |  |  |  |
| [^18^F]FE-PE2I studies (N=1) | NA |  |  |  |
| #2.2 Drug-naïve patients with MDD |  |  |  |  |
| No (N=14) | 0.11 | -0.17 – 0.39 | 56% | 0.455 |
| Yes (N=3) | NA |  |  |  |
| #2.3 Treatment-resistant patients with MDD |  | | | |
| No (N=16) | -0.21 | -0.76 – 0.32 | 90% | 0.428 |
| Yes (N=1) | NA |  |  |  |
